# Complementary CRISPR genome-wide genetic screens in PARP10-knockout and overexpressing cells identify synthetic interactions for PARP10-mediated cellular survival

**DOI:** 10.18632/oncotarget.28277

**Published:** 2022-09-28

**Authors:** Jude B. Khatib, Emily M. Schleicher, Lindsey M. Jackson, Ashna Dhoonmoon, George-Lucian Moldovan, Claudia M. Nicolae

**Affiliations:** ^1^Department of Biochemistry and Molecular Biology, The Pennsylvania State University College of Medicine, Hershey, PA 17033, USA; ^*^These authors contributed equally to this work

**Keywords:** PARP10, ATM, CRISPR screens, genome stability, cancer cell proliferation

## Abstract

PARP10 is a mono-ADP-ribosyltransferase with multiple cellular functions, including proliferation, apoptosis, metabolism and DNA repair. PARP10 is overexpressed in a significant proportion of tumors, particularly breast and ovarian cancers. Identifying genetic susceptibilities based on PARP10 expression levels is thus potentially relevant for finding new targets for precision oncology. Here, we performed a series of CRISPR genome-wide loss-of-function screens in isogenic control and PARP10-overexpressing or PARP10-knockout cell lines, to identify genetic determinants of PARP10-mediated cellular survival. We found that PARP10-overexpressing cells rely on multiple DNA repair genes for survival, including ATM, the master regulator of the DNA damage checkpoint. Moreover, we show that PARP10 impacts the recruitment of ATM to nascent DNA upon replication stress. Finally, we identify the CDK2-Cyclin E1 complex as essential for proliferation of PARP10-knockout cells. Our work identifies a network of functionally relevant PARP10 synthetic interactions, and reveals a set of factors which can potentially be targeted in personalized cancer therapy.

## INTRODUCTION

The PARP family of enzymes contains at least 17 enzymes with PARP (poly-ADP-ribose polymerase) domains in their C-termini. This domain catalyzes the conjugation of ADP-ribose moieties to substrate proteins [1–3]. PARP1, the founding member of the family, and a number of other PARP family members catalyze the formation of poly-ADP-ribose chains. In contrast, a subset of PARP family members only catalyze the transfer of a single ADP-ribose molecule (a process known as mono-ADP-ribosylation, or MARylation) [4]. PARP10 (also known as ARTD10) is such a mono-ADP-ribosyltransferase. The functions of PARP10 appear to be distinct than those of PARP1, and its catalytic activity is not affected by PARP1 inhibitors, which have been recently approved for treatment of breast and ovarian tumors with BRCA mutations [5, 6]. PARP10 was initially identified as a Myc-interacting protein [7]. Subsequently, potential roles for PARP10 in cell cycle transition [8], apoptosis [9], NFkB pathway [10], mitochondrial oxidation [11], cell migration [12], and neuronal excitability [13] have been described. For most of these functions, molecular mechanisms are still unclear, and it is generally not known if PARP10-catalyzed MARylation is involved in all of these processes.

Proliferating cells are exposed to replication stress, defined as the arrest of the replication machinery and formation of aberrant replication structures upon encountering of obstacles to DNA polymerases (such DNA lesions, fragile sites, secondary DNA structures or transcription bubbles) [14]. One mechanism that restarts stalled replication forks is translesion synthesis (TLS), which allows the DNA replication machinery to bypass DNA lesions. This occurs through the action of specialized, error-prone polymerases which are recruited to stalled replication forks upon ubiquitination of PCNA, a replication fork component which serves as a co-factor for DNA polymerases [15–19].

We previously showed that PARP10 may be involved in regulating TLS [20]. We found that PARP10 interacts with ubiquitinated PCNA, is required for maintaining PCNA ubiquitination levels, and promotes TLS-dependent mutagenesis. More recently [21], we created PARP10-knockout HeLa cells using CRISPR and found that they have increased sensitivity to hydroxyurea (HU), a drug that induces replication arrest by depleting nucleotide pools. Conversely, PARP10-overexpressing cells were resistant to HU. These results suggested that PARP10 participates in alleviating replication stress by promoting TLS. Finally, by mining publicly available cancer datasets, we showed that PARP10 is overexpressed in about a third of all ovarian tumors and a fifth of all breast tumors. We proposed that PARP10 overexpression during transformation allows suppression of replication stress through TLS-mediated bypass of replication arresting structures, thereby allowing hyper-proliferation of cancer cells.

In recent years, genome-wide CRISPR genetic screens have emerged as powerful tools for identifying clinically-relevant genetic interactions, such as synthetic lethality interactions, as well as genetic biomarkers of drug response [22, 23]. Here, we employed complementary CRISPR loss-of-function genome-wide screening to identify genes required for proliferation of PARP10-overexpressing and PARP10-knockout cells. We found that DNA repair factors, including ATM, a master regulator of the DNA damage checkpoint response, are specifically promoting the proliferation of PARP10-overexpressing cells. Moreover, we identified a role for PARP10 in regulating ATM recruitment to stressed replication forks. Finally, we found that the CDK2-cyclin E1 complex is specifically required for the proliferation of PARP10-deficient cells. Our work reveals novel PARP10 genetic interactions of functional relevance and identifies a set of factors which can potentially be targeted in personalized cancer therapy.

## RESULTS

### Genome-wide CRISPR knockout screens to identify genes required for viability of PARP10-overexpressing breast cells

**Figure 1:**
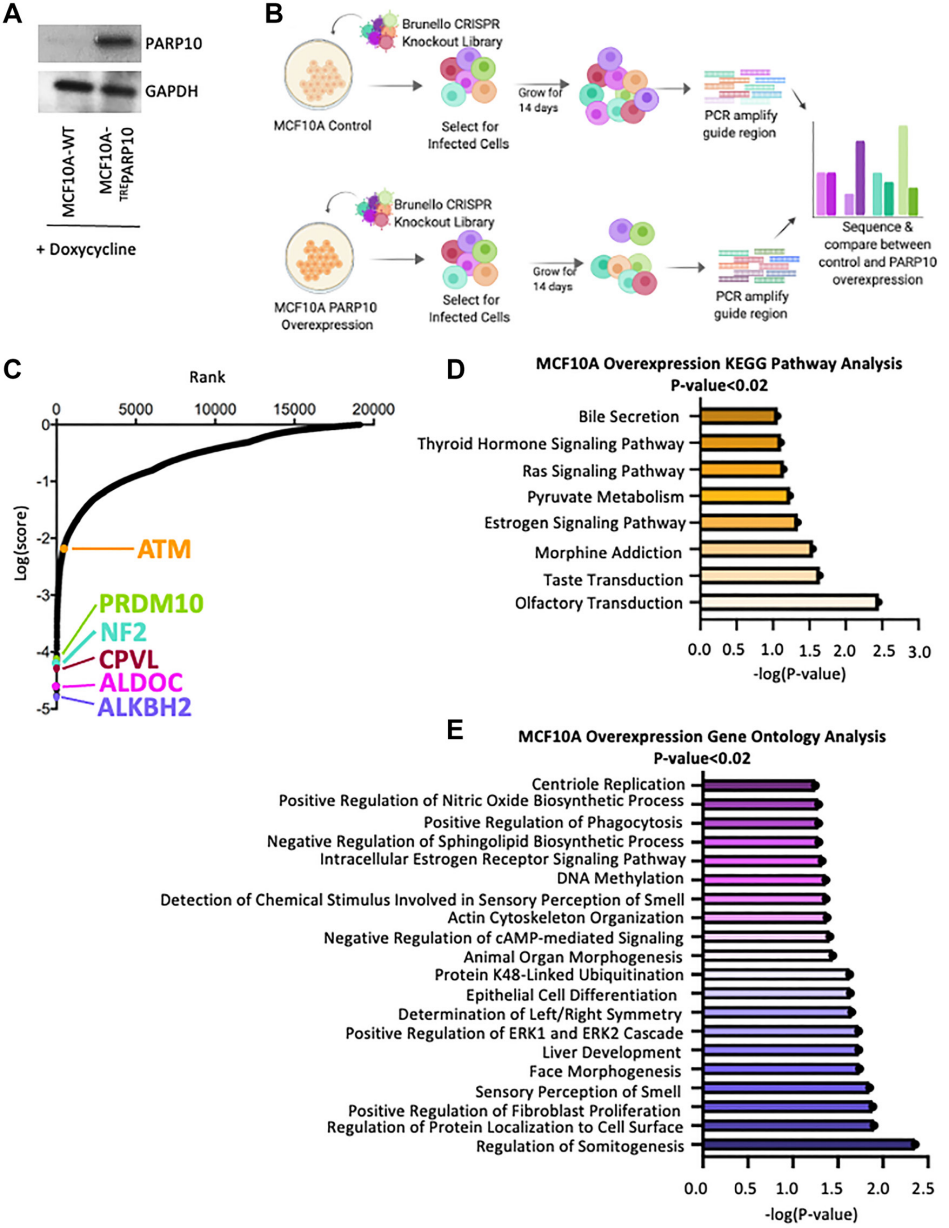
Identification of genes necessary for proliferation of PARP10-overexpressing MCF10A breast epithelial cells by CRISPR-mediated genome-wide loss-of-function screening. (**A**) Western blot showing doxycycline-induced overexpression of PARP10 in MCF10A cells. (**B**) Overview of the CRISPR knockout screens to identify genes that are specifically required for proliferation of PARP10-overexpressing MCF10A cells. (**C**) Scatterplot showing the results of genome-wide CRISPR knockout screens to identify genes that are specifically required for proliferation of PARP10-overexpressing MCF10A cells. Each gene targeted by the library was ranked according to the MAGeCK score indicating genes which, when inactivated, specifically cause reduced proliferation in PARP10-overexpressing MCF10A-^TRE^PARP10 cells compared to control MCF10A cells. Top hits chosen for validation are indicated. (**D**, **E**) Biological pathway analyses using KEGG (D) or Gene Ontology (E) analyses of the top hits with *p*-values lower than 0.02 which specifically cause reduced proliferation in PARP10-overexpressing MCF10A-^TRE^PARP10 cells compared to control MCF10A cells. KEGG terms with negative logP greater than 1 are shown. GO_BP terms with negative logP greater than 1.24 are presented (corresponding to the top 20 pathways).

By analyzing publicly-available TCGA datasets, we previously showed that PARP10 is overexpressed in about a third of all ovarian tumors and a fifth of all breast tumors [21]. We thus sought to identify genetic determinants of cellular viability upon PARP10 overexpression, in the hope of identifying new targets for precision treatment of PARP10-overexpressing tumors. Since PARP10 is mostly overexpressed in breast or ovarian tumors, we first created an isogenic PARP10 overexpression genetic system by exogenously expressing PARP10 under the doxycycline-inducible TRE promoter in the non-cancer breast epithelial cell line MCF10A (Figure 1A). We speculate that this mimics the situation in breast cancer patients, with the normal breast tissue having normal PARP10 expression, and the breast cancer cells overexpressing PARP10. We next infected the control (parental) MCF10A cell line and the cell line harboring the PARP10-expressing system (MCF10A-^TRE^PARP10) with the Brunello genome-wide CRISPR-knockout lentiviral library [24], which targets 19,114 human genes with an average of 4 guide RNAs (gRNAs) for each gene, for a total of 76,441 unique gRNAs. Taking care to maintain 250x fold library coverage at all times (equivalent to 20 million cells), we grew library-infected cells for two weeks in the presence of doxycycline (Figure 1B). Cells were then collected, and genomic DNA was extracted. The gRNA region was amplified by PCR and identified by Illumina sequencing. Bioinformatic analyses using the MAGeCK algorithm [25] were used to generate ranking lists of genes that were lost in MCF10A-^TRE^PARP10 cells compared to parental MCF10A cells (Figure 1C, Supplementary Table 1). This represents genes which, when inactivated, result in death of PARP10-overexpressing MCF10A cells, but not of normal MCF10A cells.

**Figure 2:**
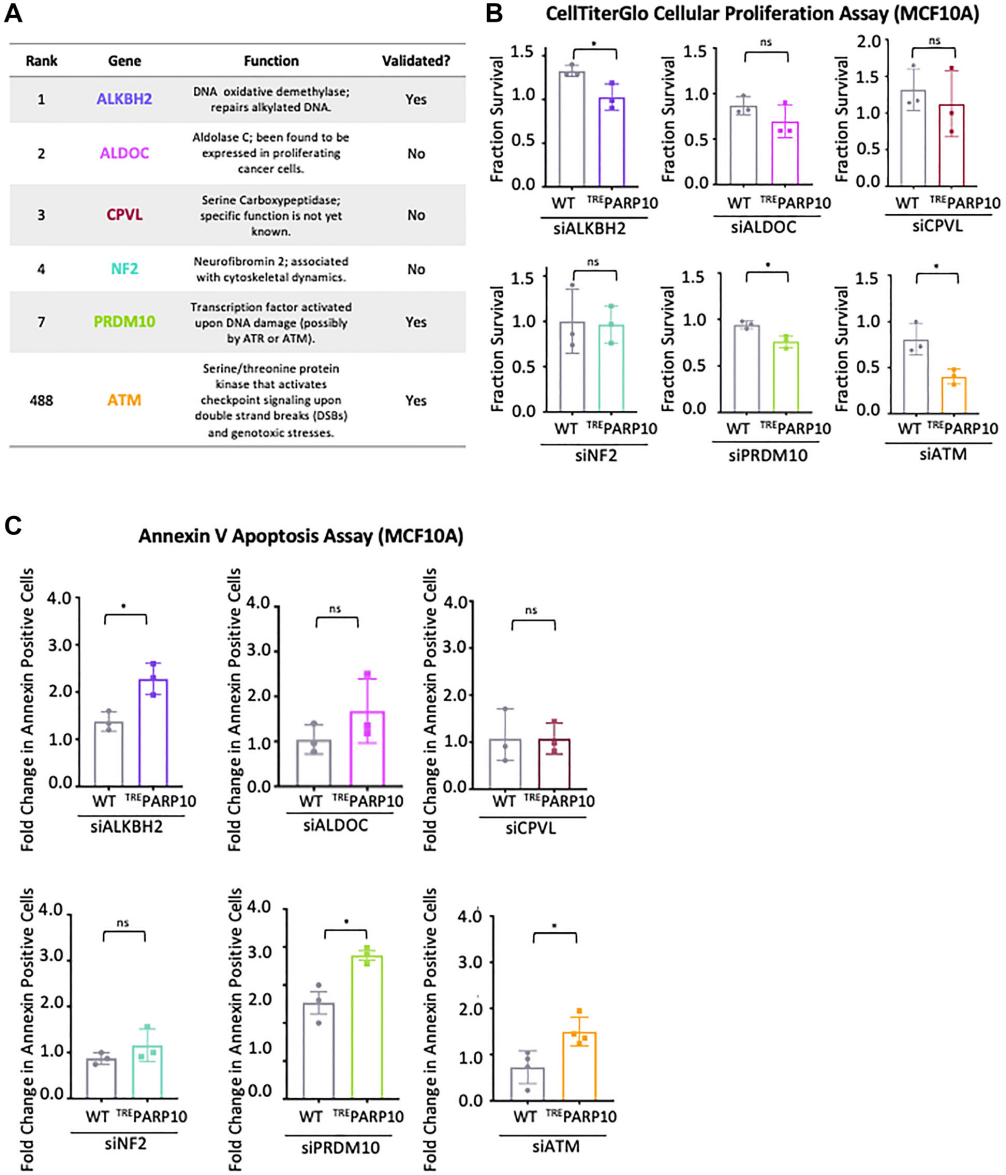
Validation of the top hits from the CRISPR screen for genetic determinants of proliferation of PARP10-overexpressing cells. (**A**) Table showing the screen ranking, and the biological functions of the hits chosen for subsequent validation. Also indicated is whether the hits could be validated in subsequent experiments or not. (**B**) CellTiterGlo cellular proliferation assays showing that knockdown of ALKBH2, PRDM10 or ATM reduces proliferation of PARP10-overexpressing MCF10A-^TRE^PARP10 cells compared to control MCF10A cells. In contrast, no statistically significant impact was observed for ALDOC, CPVL or NF2. The average of three experiments is shown (normalized to control siRNA). Error bars represent standard deviations, and asterisks indicate statistical significance (*t*-test, two-tailed, unpaired). Western blots or qRT-PCR experiments confirming the knockdowns are shown in Supplementary Figure 1A, 1B. (**C**) Annexin V assays showing that knockdown of ALKBH2, PRDM10 or ATM results in increased apoptosis in PARP10-overexpressing MCF10A-^TRE^PARP10 cells compared to control MCF10A cells. In contrast, no statistically significant impact was observed for ALDOC, CPVL or NF2. The average of three experiments (or four for siATM) is presented (normalized to control siRNA), with standard deviations shown as error bars. Asterisks indicate statistical significance (*t*-test two-tailed, unpaired).

We next performed biological pathway enrichment analyses of the top screen hits (with *p*-values lower than 0.02), using both KEGG and Gene Ontology databases. With a few exceptions (regulation of ERK signaling, regulation of fibroblast proliferation), there were no enriched biological pathways directly relevant to cell survival or proliferation (Figure 1D, 1E). We thus decided to attempt to validate the topmost four hits, namely ALKBH2 (dioxygenase involved in the direct repair of methylated adenines and cytosines), ALDOC (Aldolase C, involved in glucose metabolism), CPVL (carboxypeptidase of unknown function) and NF2 (Neurofibromin-2, involved in cytoskeletal dynamics) (Figure 2A). Since we previously identified a role for PARP10 in replication stress tolerance, and showed that PARP10-deficient cells are sensitive to replication stress while PARP10-overexpressing cells are resistant to it [20, 21] we searched if, in addition to the top ranked hit ALKBH2, there are any other DNA repair associated genes ranked as top hits. We found that PRDM10, a transcription factor with unknown factors but which is potentially phosphorylated by the DNA damage checkpoint kinases ATM and ATR [26] ranked 7th, while ATM [27–31] itself ranked 488th (corresponding to the top 2.5%). We thus included PRDM10 and ATM in the list of genes chosen for validation (Figure 2A).

To validate these six hits, we employed specific siRNA oligonucleotides to knock down their expression in MCF10A-^TRE^PARP10 cells or control MCF10A cells. Gene knockdown was confirmed by western blot or quantitative RT-PCR (Supplementary Figure 1A, 1B). We next measured cellular proliferation using the CellTiterGlo assay. Three of the hits, namely ALKBH2 (ranked 1st), PRDM10 (ranked 7th) and ATM (ranked 488th) showed a significantly higher reduction in cellular proliferation when knocked down in MCF10A-^TRE^PARP10 cells compared to control MCF10A cells (Figure 2B). These findings indicate that these genes are specifically required for proliferation of PARP10-overexpressimg cells compared to control cells, thus validating our CRISPR screen. In contrast, we did not observe a differential impact on proliferation of MCF10A-^TRE^PARP10 cells compared to control MCF10A cells upon knockdown of ALDOC (ranked 2nd), CPVL (ranked 3rd) or NF2 (ranked 4th) (Figure 2B). These findings suggest that, even though these genes scored highly in our CRISPR screen, they may not differentially affect the proliferation of PARP10-overexpression cells.

Next, we also measured the impact of the knockdown of these six hits on apoptosis, as measured by the Annexin V assay. In line with the cellular survival results described above, knockdown of ALKBH2, PRDM10 and ATM resulted in significantly higher apoptosis in MCF10A-^TRE^PARP10 cells compared to control MCF10A cells, while knockdown of ALDOC, CPVL and NRF2 did not (Figure 2C). Of note, knockdown of ALDOC (ranked 2nd) did show a trend towards increased impact in MCF10A-^TRE^PARP10 cells, in both cellular proliferation and apoptosis assays, suggesting that it may represent a true hit, but this trend was not statistically significant. In conclusion, we were able to validate at least three top hits out the six hits investigated, by showing that their knockdown specifically impairs the proliferation and survival of PARP10-overexpressing MCF10A cells.

### ATM promotes the proliferation of PARP10-overexpressing cells

**Figure 3:**
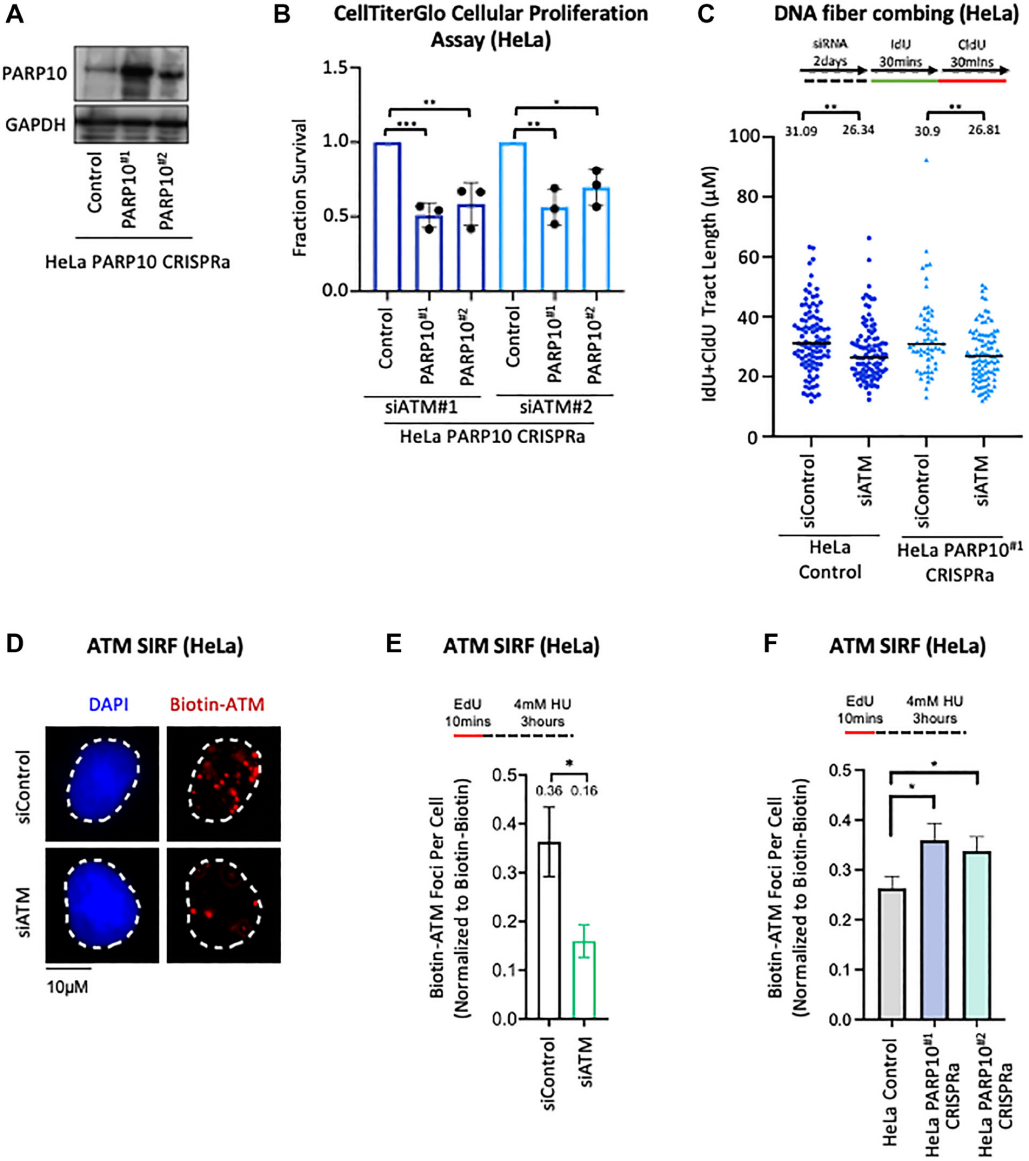
Functional interaction between ATM and PARP10 expression. (**A**) Western blot showing overexpression of PARP10 in two independent HeLa PARP10 CRISPRa cell lines. (**B**) CellTiterGlo cellular proliferation assays showing that knockdown of ATM, using two separate siRNA oligonucleotides, specifically reduces the proliferation of two different PARP10-overexpressing CRISPRa HeLa cell lines compared to control HeLa cells. The average of three experiments is shown (normalized to control siRNA). Error bars represent standard deviations, and asterisks indicate statistical significance (*t*-test, two-tailed, unpaired). (**C**) DNA fiber combing assays showing that ATM depletion does not differentially impact replication fork progression in PARP10-overexpressing CRISPRa HeLa cell lines compared to control HeLa cells. Replication tracts labeled by both IdU and CldU, indicating ongoing replication forks, were quantified, and their labeled tract length (IdU+CldU) is presented, with the median values marked on the graph and listed at the top. At least 60 tracts were quantified for each sample. Asterisks indicate statistical significance (Mann-Whitney test). A schematic representation of the assay conditions is shown at the top. (**D**–**F**) ATM SIRF experiments showing that PARP10 overexpression in HeLa cells increases HU-induced ATM binding to nascent DNA. HeLa cells were treated with 4 mM HU for 3 hours. ATM depletion was used as control, to demonstrate the specificity of the SIRF signal. Representative micrographs (D) and quantifications (E, F) are shown. Bars indicate the mean values, error bars represent standard errors, and asterisks indicate statistical significance (*t*-test, two-tailed, unpaired). Schematic representations of the assay conditions are shown at the top.

Since we previously described a putative role for PARP10 in genome stability [20, 21], and the three hits we could validate were all DNA repair-associated genes, we decided to further explore the role of DNA repair in the survival of PARP10-overexpressing cells. For this, we decided to focus on ATM, the master DNA damage checkpoint kinase [27–31]. Even though ATM was only ranked 488th in our screen, the validation experiments described above (Figure 2) showed that the impact of ATM on PARP10-overexpressing MCF10A cells was the most pronounced of the hits investigated. We first sought to investigate if the inhibitory effect of ATM depletion on PARP10-overexpressing cells is specific to MCF10A cells. To address this, we employed the CRISPR activation (CRISPRa) system to enhance the expression of PARP10, from the endogenous locus in HeLa cells. We obtained two separate clones (driven by different gRNA sequences) which showed increased PARP10 expression compared to control cells (Figure 3A). ATM knockdown significantly reduced cellular proliferation in both PARP10 CRISPRa lines compared to control (Figure 3B). In order to rule out an off-target effect of the ATM siRNA oligonucleotide employed, we also tested the impact of a second ATM siRNA oligonucleotide (labeled siATM#2). Similar to the original siRNA used (siATM#1), the second one also reduced proliferation of both PARP10 CRISPRa HeLa cell lines compared to control cells (Figure 3B). Overall, these findings show that loss of ATM specifically reduced the proliferation of PARP10-overexpressing cells in multiple cell lines.

We next sought to explore possible mechanistic connections between ATM and PARP10. Since we previously found that PARP10 interacts with the replication fork component PCNA, and may regulate replication fork progression under replication stress [20, 21], we first investigated if ATM depletion differentially impacts replication fork speed in PARP10-overexpressing cells compared to control cells. We employed the DNA fiber combing assay, which allows the quantification of the progression of individual replication forks upon treatment with thymidine analogs. While ATM depletion slightly reduced fork progression, this occurred similarly in control and PARP10-overexpressing CRISPRa HeLa cells (Figure 3C), suggesting that the inhibitory impact of ATM loss in PARP10-overexpressing cells is not caused by fork progression defects.

Next, we measured ATM recruitment to stressed replication forks by employing the SIRF (*in situ* analysis of protein interactions at replication forks) assay [32], a proximity ligation (PLA) -based approach that allows the quantification of the binding of the protein of interest to EdU-labeled nascent DNA. We observed that ATM forms SIRF foci in HU-treated HeLa cells, suggesting that ATM binds nascent DNA at stressed replication forks (Figure 3D, 3E). ATM depletion by siRNA reduced the ATM SIRF foci formation, confirming the specificity of the SIRF signal. Under the same HU treatment conditions, PARP10 overexpression resulted in an increase in ATM SIRF foci (Figure 3F), suggesting a role for PARP10 in regulating ATM binding to nascent DNA. Overall, these findings suggest that, upon replication stress, PARP10 overexpressing cells have increased activation of the ATM pathway, which may be relevant to the synthetic lethality genetic interaction observed between PARP10 overexpression and ATM loss.

### Genome-wide CRISPR knockout screens to identify genes required for viability of PARP10-knockout cells

**Figure 4:**
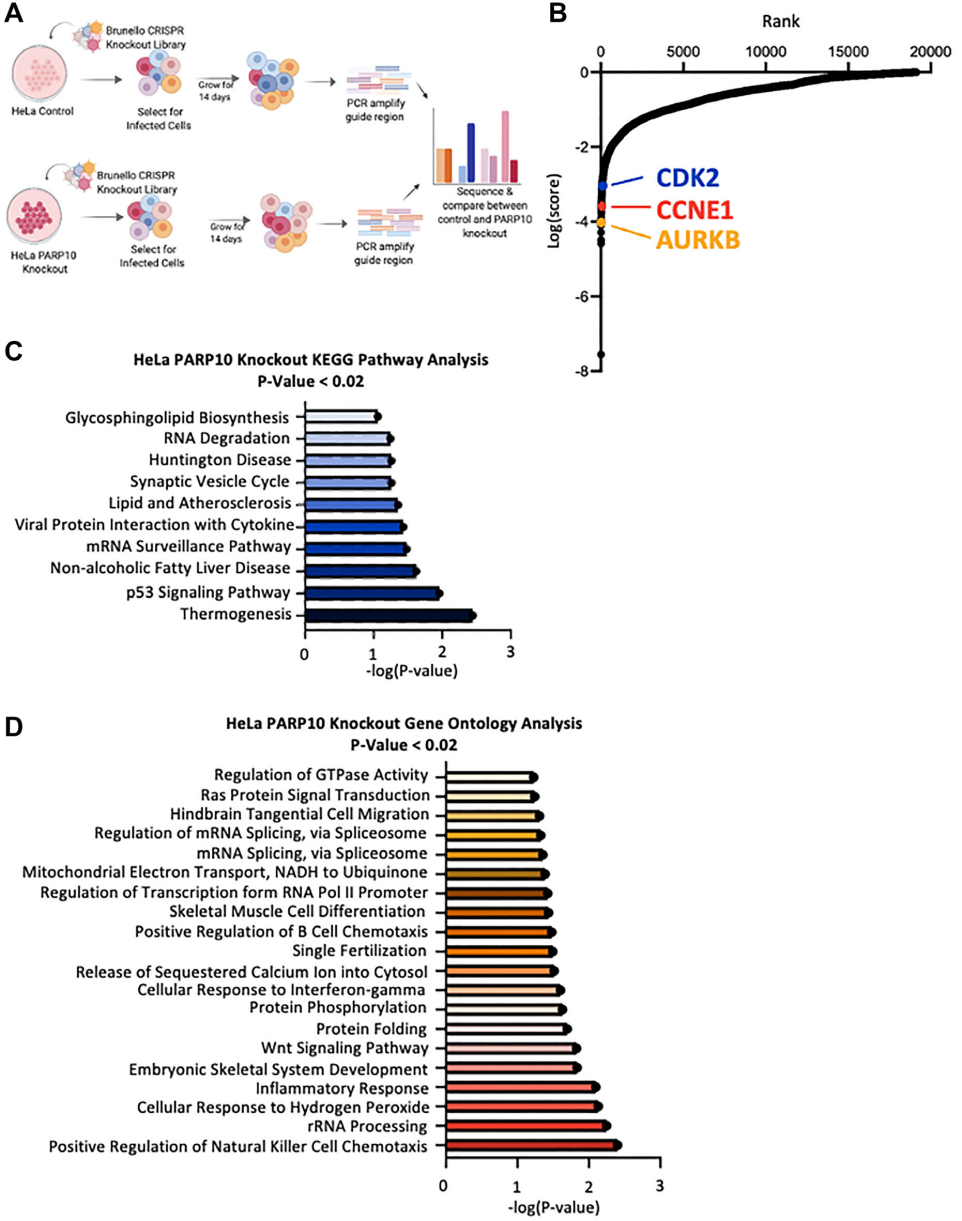
Identification of genes necessary for proliferation of PARP10-knockout HeLa cells by CRISPR-mediated genome-wide loss-of-function screening. (**A**) Overview of the CRISPR knockout screens to identify genes that are specifically required for proliferation of PARP10-knockout HeLa cells. (**B**) Scatterplot showing the results of genome-wide CRISPR knockout screens to identify genes that are specifically required for proliferation of PARP10-knockout HeLa cells. Each gene targeted by the library was ranked according to the MAGeCK score indicating genes which, when inactivated, specifically cause reduced proliferation in PARP10-knockout HeLa cells compared to control HeLa cells. Top hits chosen for validation are indicated. (**C**, **D**) Biological pathway analyses using KEGG (C) or Gene Ontology (D) analyses of the top hits with *p*-values lower than 0.02 which specifically cause reduced proliferation in PARP10-knockout HeLa cells compared to control HeLa cells. KEGG terms with negative logP greater than 1 are shown. GO_BP terms with negative logP greater than 1.22 are presented (corresponding to the top 20 pathways).

To complement the CRISPR knockout screen described above (Figure 1) in PARP10-overexpressing cells, we decided to perform a similar genome-wide CRISPR knockout screen in PARP10-knockout cells. We reasoned that, in addition to providing a complementary dataset to the PARP10-overexpression screen, the PARP10-knockout screen may also potentially uncover novel PARP10 functional insights, by identifying PARP10 synthetic lethal interactions. We also hypothesized that the potential clinical applicability of this screen goes beyond breast and ovarian cancer, since it may identify genes which can be targeted for cancer therapy in combination with PARP10 inhibitors, regardless of the PARP10 overexpression status. Therefore, for this screen, we employed the PARP10-knockout HeLa cells (HeLa-PARP10^KO^) we previously created and characterized [21]. Similar to the PARP10-overexpression screen setup described above, we infected PARP10-knockout and control (wildtype) HeLa cells with the Brunello CRISPR knockout library, and grew the library-infected cells for two weeks, taking care to maintain at least 250-fold library coverage at all times (Figure 4A).

Cells were then collected, and genomic DNA was extracted. The gRNA region was amplified by PCR and identified by Illumina sequencing. Bioinformatic analyses using the MAGeCK algorithm were used to generate ranking lists of genes that were lost in HeLa-PARP10^KO^ cells compared to control HeLa cells (Figure 4B, Supplementary Table 2). This represents genes which, when inactivated, result in death of PARP10-knockout HeLa cells, but not of normal HeLa cells. We next performed biological pathway enrichment analyses of the top screen hits (with *p*-values lower than 0.02), using both KEGG and Gene Ontology databases (Figure 4C, 4D). The p53 pathway showed up as a top biological process enriched, suggesting that this pathway may control the survival of PARP10-deficient cells.

**Figure 5:**
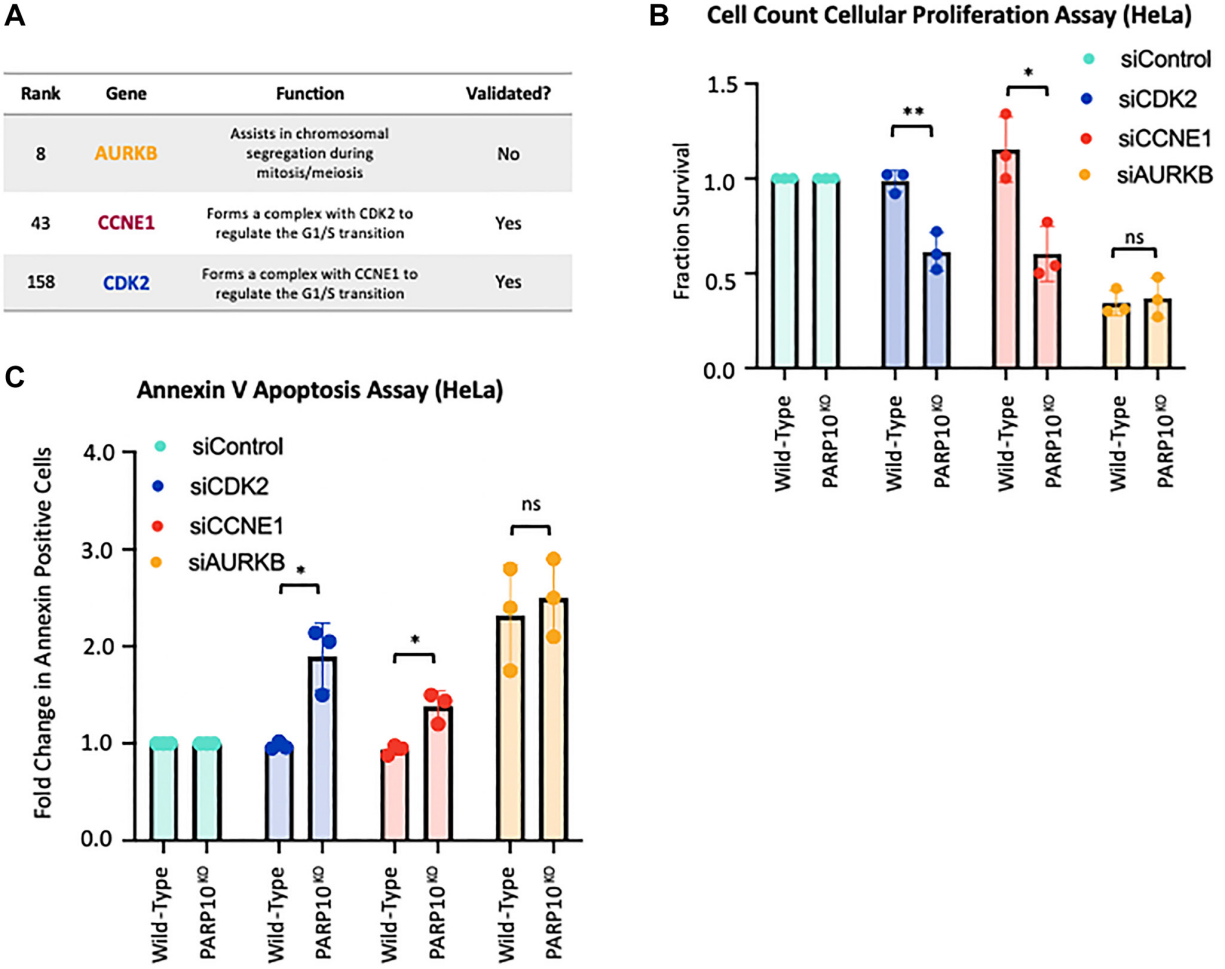
Validation of the top hits from the CRISPR screen for genetic determinants of proliferation of PARP10-knockout cells. (**A**) Table showing the screen ranking, and the biological functions of the hits chosen for subsequent validation. Also indicated is whether the hits could be validated in subsequent experiments or not. (**B**) Cell count cellular proliferation assays showing that knockdown of CDK2 or CCNE1 reduces proliferation of PARP10-knockout HeLa cells compared to control HeLa cells. In contrast, no statistically significant impact was observed for AURKB. The average of three experiments is shown (normalized to control siRNA). Error bars represent standard deviations, and asterisks indicate statistical significance (*t*-test, two-tailed, unpaired). Western blots confirming the knockdowns are shown in Supplementary Figure 1C. (**C**) Annexin V assays showing that knockdown of CDK2 or CCNE1 reduces viability of PARP10-knockout HeLa cells compared to control HeLa cells. In contrast, no statistically significant impact was observed for AURKB. The average of three experiments is presented (normalized to control siRNA), with standard deviations shown as error bars. Asterisks indicate statistical significance (*t*-test two-tailed, unpaired).

When inspecting the top hits clustering in the p53 pathway, we noticed the presence of the CDK2-Cyclin E1 complex (CCNE1, ranked 43rd; CDK2, ranked 158th), which has been previously shown to interact with PARP10 [8]. We thus picked these hits for validation. In addition we also sought to validate another top-ranked cell cycle regulator, namely Aurora B (AURKB, ranked 8th) (Figure 5A). We employed siRNA to knock-down CDK2, CCNE1, and AURKB in HeLa-PARP10^KO^ and control cells. Western blot experiments confirmed the knockdown (Supplementary Figure 1C–1E). Cellular proliferation experiments showed that depletion of CDK2 and CCNE1 significantly reduced the proliferation of PARP10-knockout cells compared to control cells (Figure 5B), thus validating our screen and suggesting that the CDK2-Cyclin E1 promotes the survival of PARP10-knockout cells. In line with this, annexin V experiments indicated that depletion of CDK2 or of CCNE1 resulted in a significantly higher increase in apoptosis in HeLa-PARP10^KO^ cells compared to control cells (Figure 5C). In contrast, depletion of AURKB did not preferentially affect the proliferation of apoptosis induction of PARP10-knockout cells compared to control cells (Figure 5B, 5C), despite AURKB scoring as a top hit in the screen. In conclusion, we could validate two of the three hits from the CRISPR PARP10-knockout synthetic lethality screen that we tested.

## DISCUSSION

Genome-wide CRISPR genetic screening has emerged as a powerful tool for revealing novel functions of genes, as well as for identifying clinically-relevant genetic interactions such as the discovery of genetic markers of sensitivity or resistance to novel therapeutic drugs [22, 23]. In this work, we employed complementary CRISPR genome-wide genetic loss-of-function screening to identify genes required for proliferation of PARP10-overexpressing and PARP10-knockout cells.

For the PARP10-overexpressing screen (Figure 1), we decided to employ the non-cancer breast epithelial cell line MCF10A. Since PARP10 is overexpressed in a significant proportion of breast and ovarian cancer tumors, we reasoned that our screening setup may mimic the situation in cancer patients, with PARP10 being overexpressed in the tumor cells but not in the normal tissue. Thus, this screen should allow us the identification of genes specifically promoting the proliferation of PARP10-overexpressing cells compared to normal cells expressing endogenous PARP10 levels. These genes may potentially represent novel targets for personalized breast and ovarian cancer therapy, since their targeting should only inhibit proliferation of PARP10-overexpressing tumor cells, but should not affect the survival of normal tissues.

For validation experiments of the PARP10-overexpression screen, we picked six of the top hits: ALKBH2, ALDOC, CPVL, NF2, PRDM10 and ATM. We were able to validate only three of the six hits (ALKBH2, PRDM10 and ATM) (Figure 2). This may indicate that a significant proportion of the hits in this screen (and potentially in CRISPR screens in general) are false positives, potentially caused by off-target effects. Indeed, this may be expected for any genome-wide screen [33–36]. On the other hand, we cannot rule out that our inability to validate three of the six hits (ALDOC, CPVL and NF2) reflects an incomplete depletion of these three proteins by siRNA (as opposed to the complete loss of function in the CRISPR screen), with the remaining protein being enough to allow proliferation of PARP10-overexpressing cells. Perhaps in line with this, in validation experiments we found that the degree of inhibitory effect on proliferation of PARP10-overexpressing cells does not correlate with the ranking of the hits in the original screen: depletion of the relatively low-ranking ATM (ranked 488th) has a stronger inhibitory effect compared to depletion of the high-ranking ALKBH2 (ranked 1st) and PRDM10 (ranked 7th) (Figure 2B, 2C). Moreover, depletion of one of the other three genes (namely ALDOC) did show a trend towards inhibitory effect in PARP10-overexpressing cells, but which was not statistically significant (Figure 2B, 2C).

Interestingly, the three genes we validated (ALKBH2, PRDM10 and ATM) are all linked to DNA repair process [26–31, 37] (Figure 2A). This may suggest that DNA repair processes are essential for proliferation of PARP10-overexpressing cells -with the caveat that pathway analyses did not identify DNA repair processes as enriched within the top hits (Figure 1D, 1E). Nevertheless, these results are perhaps in line with our previously-published findings that PARP10 is involved in genomic stability through PCNA-mediated replication fork dynamics [20, 21].

One of the most potent hits we validated is ATM, the master DNA damage checkpoint kinase [27–31]. While we found that ATM depletion reduced viability of PARP10-overexpressing cells, it remains to be seen if its pharmacological inhibition causes a similar reduction. In an attempt to identify mechanistic connections between PARP10 and ATM, we unexpectedly found that PARP10 expression correlates with ATM loading on nascent DNA upon replication stress (Figure 3D–3F). ATM was previously shown to be recruited to stressed replication forks, and promote their stabilization and repair [38–41]. Overall, our findings suggest that PARP10-overexpressing cells may be hyper-reliant on ATM activation for maintaining viability. On the other hand, it is also possible that PARP10 may directly regulate ATM recruitment. Either way, we speculate that this mechanistic connection between PARP10 expression and ATM recruitment to stressed replication forks may contribute to the genetic interaction observed. Our results also suggest that the p53 status is not relevant for the reduced viability observed upon ATM depletion in PARP10-overexpressing cells, since unlike MCF10A cells, p53 levels are low in HeLa cells, but the impact of ATM depletion was similar in the two cell lines.

For the PARP10-knockout synthetic lethality screen, we employed the HeLa-PARP10^KO^ cell line we previously created and characterized [21]. Since PARP10 inhibitors are currently being developed [42–45], we reasoned that this screen may result in the identification of new targets for cancer therapy in combination with PARP10 inhibition, regardless of the PARP10 overexpression status. Thus, the potential clinical applicability of this screen goes beyond breast and ovarian cancers. As with the PARP10-overexpressing screen, we were not able to validate all hits tested: we were able to confirm that depletion of CDK2 and CCNE1 specifically reduces the viability of PARP10-knockout cells, but we did not find a significant difference for AURKB (Figure 5). The identification of the CDK2-Cyclin E1 complex is of particular relevance, since this complex was shown to phosphorylate PARP10 *in vitro*, and this phosphorylation was suggested to be functionally relevant for cell cycle progression [8]. Our results suggest that inhibition of this complex may be combined with PARP10 inhibition for reducing the proliferation of cancer cells. In this screen, ATM did not show up as a hit, suggesting that it plays a role in PARP10-overexpressing cells but not in PARP10-deficient cells.

## MATERIALS AND METHODS

### Cell culture and protein techniques

HeLa cells were cultured in DMEM supplemented with 10% fetal calf serum and 1% Pen/Strep. MCF10A cells (ATCC CRL-10317) were cultured in DMEM/F12 supplemented with 10% FBS, 20 ng/mL hEGF, 0.5 mg/mL hydrocortisone, 100 ng/mL Cholera Toxin, 10 uG/mL insulin, and 1% Pen/Strep. HeLa-PARP10^KO^ cells were created in our laboratory and previously described [21]. For doxycycline-inducible expression of PARP10, the pLV:Bsd-TRE lentiviral construct encoding wildtype PARP10 was obtained from Cyagen, and used to infect MCF10A cells stably expressing the tetracycline transactivator (tTA) element. For induction of expression, cells were grown in the presence of 2 mg/ml doxycycline. For CRISPRa-mediated PARP10 overexpression, HeLa cells were first transduced with the dCas9 lentiviral construct (Addgene 61425-LV) and selected with 3 μg/ml blasticidin. The resulting HeLa-dCas9 cells were then transduced with the lentiviral construct for the MS2-P65-HSF1 (MPH) activator complex (Addgene 61426-LVC) and selected with 0.5 mg/ml hygromycin. Finally, HeLa-dCas9-MPH cells were transduced with lentivirus constructs containing the following guide sequences: TCAACCCCCAGCTGACCAGG for PARP10^#1^ and AATACCTCCTGGTCAGCTGG for PARP10^#2^ (Sigma-Aldrich Custom CRISPR in lentiviral backbone LV06).

Gene knockdown was performed using Lipofectamine RNAiMAX (ThermoFisher). AllStars negative control siRNA (Qiagen 1027281) was employed as control. The following oligonucleotide sequences (Stealth or SilencerSelect siRNA, ThermoFisher) were used: ALKBH2: s42494; ALDOC: s1263; CPVL: s29094; NF2: s194647; PRDM10: s32522; ATM#1: AM51331; ATM#2: s1708; CDK2: s206; CCNE1: s2524; AURKB: s17611.

Denatured whole cell extracts were prepared by boiling cells in 100 mM Tris, 4% SDS, 0.5 M β-mercaptoethanol. Antibodies used for western blots were: Vinculin (Santa Cruz sc-25336), GAPDH (Santa Cruz sc-47724), ATM (Cell Signaling 2873S), PARP10 (Novus NB100-2157), CDK2 (Santa Cruz sc-6248), CCNE1 (Cell Signaling 4129S), AURKB (Abcam ab3609).

### Cellular survival assays

For cell counting cellular proliferation assays, after 2 days of siRNA treatment, 250,000 cells were plated in 6-well plates. After 3 days, cells were counted using the EVE automated cell counter (NanoEntek), and the cell survival fraction was calculated. CellTiterGlo cellular proliferation assays were performed using the CellTiterGlo reagent (Promega G7572) according to the manufacturer’s instructions. For each condition, 1500 siRNA-treated cells were plated into 96-well plates. Three days later, CellTiterGlo reagent was added for 10 minutes and the luminescence was read on a plate reader. Apoptosis assays were performed using the FITC Annexin V kit (Biolegend, 640906). Quantification was performed using a BD FACSCanto 10 flow cytometer.

### CRISPR screens

For CRISPR knockout screens, the Brunello Human CRISPR knockout pooled lentiviral library (Addgene 73179) was used [24]. This library encompasses 76,411 gRNAs that target 19,114 genes. Fifty million cells from each cell lines (MCF10A wildtype, MCF10A-^TRE^PARP10, HeLa wildtype, HeLa-PARP10^KO^) were infected with this library at a multiplicity of infection (MOI) of 0.4 to achieve 250-fold coverage and selected for 4 days with 0.6 μg/mL puromycin. Twenty million library-infected cells (to maintain 250-fold coverage) were passaged for two weeks and then collected. Genomic DNA was isolated using the DNeasy Blood and Tissue Kit (Qiagen 69504) and employed for PCR using Illumina adapters to identify the gRNA representation in each sample. 10 μg of gDNA was used in each PCR reaction along with 20 μl 5X HiFi Reaction Buffer, 4 μl of P5 primer, 4 μl of P7 primer, 3 μl of Radiant HiFi Ultra Polymerase (Stellar Scientific), and water. The P5 and P7 primers were determined using the user guide provided with the CRISPR libraries (https://media.addgene.org/cms/filer_public/61/16/611619f4-0926-4a07-b5c7-e286a8ecf7f5/broadgpp-sequencing-protocol.pdf). The PCR cycled as follows: 98°C for 2 min before cycling, then 98°C for 10 sec, 60°C for 15 sec, and 72°C for 45 sec, for 30 cycles, and finally 72°C for 5 min. After PCR purification, the final product was Sanger sequenced to confirm that the guide region is present, followed by qPCR to determine the exact amount of PCR product present. The purified PCR product was then sequenced with Illumina HiSeq 2500 single read for 50 cycles, targeting 10 million reads. Next, the sequencing results were analyzed bioinformatically using the MAGeCk algorithm, which takes into consideration raw gRNA read counts to test if individual guides vary significantly between the conditions [25]. The MAGeCK software and instructions on running it were obtained from https://sourceforge.net/p/mageck/wiki/libraries/. Finally, analyses of the Gene Ontology pathways enriched among the top hits was performed using DAVID [46, 47].

### DNA fiber combing

Cells were treated with siRNA as indicated, for 2 days, then incubated with 100 μM IdU for 30 min, washed with PBS and incubated with 100 μM CldU for another 30 min. Cells were then harvested and processed using the FiberPrep kit (Genomic Vision EXT-001) according to the manufacturer’s instructions. DNA molecules were stretched onto coverslips (Genomic Vision COV-002-RUO) using the FiberComb Molecular Combing instrument (Genomic Vision MCS-001). Slides were incubated with primary antibodies (Abcam 6326 for detecting CIdU; BD 347580 for detecting IdU; Millipore Sigma MAB3034 for detecting DNA), washed with PBS and incubated with Cy3, Cy5 or BV480-coupled secondary antibodies (Abcam 6946, Abcam 6565 and BD Biosciences 564879). Following mounting, slides were imaged using a Leica SP5 confocal microscope. At least 60 tracts were quantified for each sample.

### *In situ* analysis of protein interactions at replication forks (SIRF)

After siRNA treatment for 2 days, HeLa cells were seeded into 8-chamber slides and 24 hours later they were pulse-labeled with 50 μM EdU for 10 min followed by 4 mM HU for 3 hr. Cells were permeabilized with 0.5% Triton for 10 min at 4C, washed with PBS, fixed at room temperature with 3.7% paraformaldehyde in PBS for 10 min, washed again in PBS, and then blocked in 3% BSA in PBS for 30 min. Cells were then subjected to Click-iT reaction with biotin-azide using the Click-iT Cell Reaction Buffer Kit (ThermoFisher, C10269) for 30 min and incubated overnight at 4C with primary antibodies diluted in PBS with 1% BSA. The primary antibodies used were: Biotin (mouse: Jackson ImmunoResearch 200-002-211; rabbit: Bethyl Laboratories A150-109A); ATM (Cell Signaling 2873S). Next, cells were subjected to a proximity ligation reaction using the Duolink kit (Millipore Sigma) according to the manufacturer’s instructions. Slides were imaged using a Leica SP5 confocal microscope and images were analyzed using ImageJ 1.52p software. At least 75 cells were quantified for each sample. For each sample, the number of ATM-biotin foci were divided by the average of the number of Biotin-Biotin foci for that respective sample.

### Quantification of gene expression by real-time quantitative PCR (RT-qPCR)

Total mRNA was purified using TRIzol reagent (Life Tech). To generate cDNA, 1 μg RNA was subjected to reverse transcription using the RevertAid Reverse Transcriptase Kit (Thermo Fisher Scientific) with oligo-dT primers. Real-time qPCR was performed with PerfeCTa SYBR Green SuperMix (Quanta), using a CFX Connect Real-Time Cycler (BioRad). The cDNA of GAPDH gene was used for normalization. Primers used were: ALKBH2 for: GACTGGACAGACCTTCAAC, ALKBH2 rev: AGGAGACAGAGGCAATGG [48]; CPVL for: TCAACCTGAACGGAATTGCTA, CPVL rev: GAAGGATCACTTGTTAAGTCGC [49]; ALDOC for: ATGCCTCACTCGTACCCAG, ALDOC rev: TTTCCACCCCAATTTGGCTCA [50]; NF2 for: CCCCCAACTCCCCTTTCC, NF2 rev: AGCCCTTTAGCCCCCCTG [51]; PRDM10 for: GTGAAAAAACACGTGCGC, PRDM10 rev: ACACAGGAAGTCTTTGCG; GAPDH for: AAATCAAGTGGGGCGATGCTG, GAPDH rev: GCAGAGATGATGACCCTTTTG.

### Statistical analyses

For the cellular survival assays and SIRF assays, the *t*-test (two-tailed, unpaired) was used. For the DNA fiber assay, the Mann-Whitney statistical test was performed. Statistical significance is indicated for each graph (ns = not significant, for *P* > 0.05; ^*^ for *P* ≤ 0.05; ^**^ for *P* ≤ 0.01; ^***^ for *P* ≤ 0.001, ^****^ for *P* ≤ 0.0001). The MAGeCK files showing the complete CRISPR screening datasets are presented in the Supplementary Tables 1 and 2. All source data underlying each of the figures, including the values plotted in graphs, the exact *p*-values, and the uncropped blots are presented in the Supplementary Table 3.

## SUPPLEMENTARY MATERIALS








