# Supplementary material for: Complementary CRISPR genome-wide genetic screens in PARP10-knockout and overexpressing cells identify synthetic interactions for PARP10-mediated cellular survival
Source: Oncotarget. 2022 Sep 28;13:1078–91. doi: 10.18632/oncotarget.28277 (PMC9518689; doi:10.18632/oncotarget.28277)
Supplement: Supplementary file 1 [file oncotarget-13-28277-s001.pdf]

Complementary CRISPR genome-wide genetic screens in PARP10-knockout and overexpressing cells identify synthetic interactions for PARP10-mediated cellular survival

SUPPLEMENTARY MATERIALS

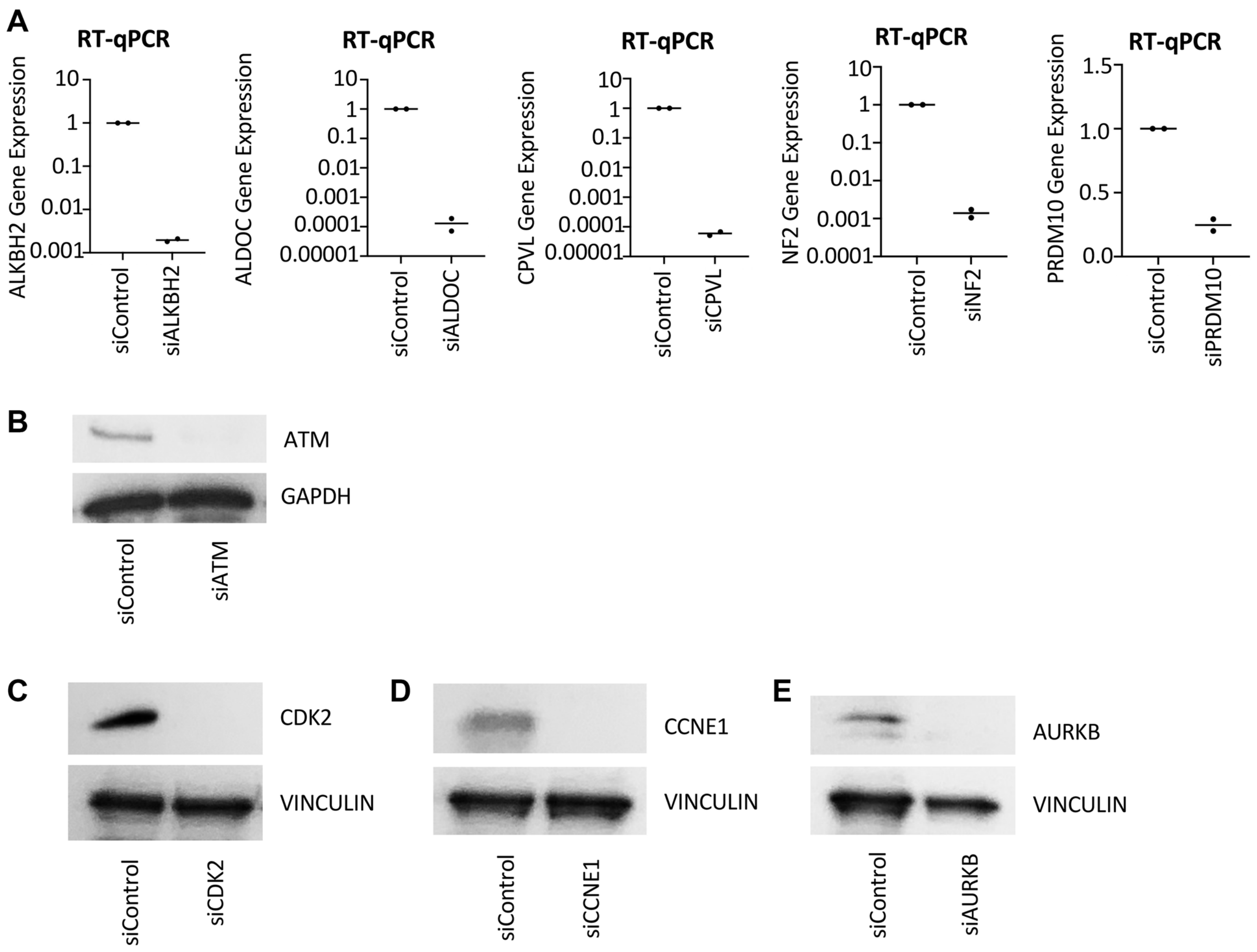

Supplementary Figure 1: Confirmation of gene knockdowns. (**A**) RT-qPCR experiment showing reduction in mRNA levels for ALKBH2, ALDOC, CPVL, NF2 and PRDM10 upon siRNA-mediated knockdown. The average of two technical replicates is shown. (No antibody was available to us for verifying the depletion by Western blot.) (**B–E**) Western blots showing the depletion of ATM (B), CDK2 (C), CCNE1 (D) and AURKB (E) upon siRNA treatment in HeLa cells.

Supplementary Table 1: MAGeCK analyses of the CRISPR screens identifying genes necessary for proliferation of PARP10-overexpressing MCF10A breast epithelial cells. Supplementary Table 1

Supplementary Table 2: MAGeCK analyses of the CRISPR screens identifying genes necessary for proliferation of PARP10-knockout HeLa cells. Supplementary Table 2

Supplementary Table 3: The source data underlying each of the figure panels, including: the values plotted in graphs, the exact *p*-values, and the uncropped blots. Supplementary Table 3
